# Ultra-Bright Near-Infrared Perovskite Light-Emitting Diodes with Reduced Efficiency Roll-off

**DOI:** 10.1038/s41598-018-33729-9

**Published:** 2018-10-19

**Authors:** Antonella Giuri, Zhongcheng Yuan, Yanfeng Miao, Jianpu Wang, Feng Gao, Nicola Sestu, Michele Saba, Giovanni Bongiovanni, Silvia Colella, Carola Esposito Corcione, Giuseppe Gigli, Andrea Listorti, Aurora Rizzo

**Affiliations:** 1 Istituto di Nanotecnologia CNR-Nanotec, Distretto Tecnologico via Arnesano 16, 73100 Lecce, Italy; 2 Dipartimento di Ingegneria dell’Innovazione, Università del Salento, Via per Arnesano, 73100 Lecce, Italy; 3 Biomolecular and Organic Electronics, IFM, Linköping University, 58183 Linköping, Sweden; 4 Key Laboratory of Flexible Electronics (KLOFE) & Institute of Advanced Materials (IAM), Jiangsu National Synergetic Innovation Center for Advanced Materials (SICAM), Nanjing Tech University (Nanjing Tech), 30 South Puzhu Road, Nanjing, 211816 China; 5 Dipartimento di Fisica, Università degli Studi di Cagliari, I-09042 Monserrato, Italy; 6 Dipartimento di Matematica e Fisica “E. De Giorgi”, Università del Salento, Via per Arnesano, 73100 Lecce, Italy

## Abstract

Herein, an insulating biopolymer is exploited to guide the controlled formation of micro/nano-structure and physical confinement of α-δ mixed phase crystalline grains of formamidinium lead iodide (FAPbI_3_) perovskite, functioning as charge carrier concentrators and ensuring improved radiative recombination and photoluminescence quantum yield (PLQY). This composite material is used to build highly efficient near-infrared (NIR) FAPbI_3_ Perovskite light-emitting diodes (PeLEDs) that exhibit a high radiance of 206.7 W/sr*m^2^, among the highest reported for NIR-PeLEDs, obtained at a very high current density of 1000 mA/cm^2^, while importantly avoiding the efficiency roll-off effect. In depth photophysical characterization allows to identify the possible role of the biopolymer in *i)* enhancing the radiative recombination coefficient, improving light extraction by reducing the refractive index, or *ii)* enhancing the effective optical absorption because of dielectric scattering at the polymer-perovskite interfaces. Our study reveals how the use of insulating matrixes for the growth of perovskites represents a step towards high power applications of PeLEDs.

## Introduction

Halide perovskites are currently explored as active materials in light-emitting diodes (LEDs) with very promising outcomes^1,2^. Some of the optoelectronic properties of perovskites are ideal for efficient LEDs, particularly the color-tunable emission, the easy manufacturing^1,3,4^, and the reduced formation of active electronic trap states^5,6^. However, some of the peculiar characteristics of three-dimensional hybrid halide perovskites such as loosely bound excitons and very high carrier mobilities, instead, hinder electroluminescence. At room temperature, excitons in hybrid halide perovskites have indeed low binding energies^7,8^, therefore PLQY increases at high excitation flux when radiative bimolecular recombination dominates. In the actual PeLEDs, radiative recombination competes with trap-mediated non-radiative decay and Auger recombination mechanisms, the latter being the principal factors responsible of the device efficiency roll-off at high current density regimes^5,6,9,10^. In general, the term “efficiency roll off” is used to describe the decreasing of the efficiency of the device at high current density regimes. It can be quantified by the critical current density, which represents the current density at which the external quantum efficiency (EQE) drops to half of its maximum value^11^. In perovskite LEDs, the origin of this phenomenon is still controversial, but it has been recently attributed to either non-radiative processes that reduce the luminescence efficiency or to excessive population of charge carriers passing through the device without forming electron-hole pairs^12^. Spatial confinement of injected carries has been proven to enhance radiative recombination at low excitation levels, then improve the performance of Pe-LEDs^1,6,13–15^. The simplest approach to promote the spatial confinement of the injected charges devises the growth of ultra-thin perovskite active layer (~20 nm), which however easily leads to inhomogeneous substrate coverage causing current leakages and shorts^14,16^. Alternatively, the confinement effect has been achieved by using low-dimensional perovskite nanocrystals or by growing multiple quantum wells mixing 2D/3D perovskites^1,13–15,17–19^. At high current densities, however, the increased spatial confinement tends to increase Auger recombination rates, so that strategies that boost emission of PeLEDs at low current densities end up in causing an efficiency roll-off at high currents^12^. Preventing this phenomenon is of paramount importance and presently only few work have tried to address it, among those the most successful strategy resulted to be the tuning of the quantum well width, in 2D/3D, leading to a reduction in the local carrier density^12^.

In this work, we report a simple method to engineer the phase composition and to finely tune the grain sizes of perovskite with the precise scope of sustaining high radiance at elevated current density regimes, thus restraining the roll-off effect. This was achieved by using starch biopolymer as templating agent for the growth of formamidinium lead iodide (NH_2_CH_2_NH_3_PbI_3_ or FAPbI_3_) 3D perovskite grains^20,21^. Starch is organized into linear or branched structures defined amylose and amylopectin, respectively, composed by repeating a-D-glucopyranosyl units (Fig S1)^22,23^. Thanks to the presence of –OH groups through the molecular chains^20,21^, starch interacts with perovskite components in the precursor solution and guides the formation of uniform polycrystalline perovskite films with perfect surface coverage and well-interconnected small grains with size down to 100 nm.

Near-infrared LEDs, exploiting the peculiar prerogatives of perovskite FAPbI_3_/starch active layer, was successfully developed reaching an impressive radiance value of 206.7 W/sr*m^2^ obtained at very high currents density of ~1000 mA/cm^2^, a value never yet reported for IR emitting PeLEDs^12^. Perovskite micro and nano-structural changes and their influence on optoelectronic properties were studied as a function of starch content by means of an in-depth photophysical characterization confirming that the device performances are strongly related to the perovskite film structure and to the presence of the insulating polymer. Though X-ray diffraction we found that starch leads to the formation of mixed domains of α (emissive) and δ (non-emissive) FAPbI_3_ phases, a crystalline scenario that have been proven to reduce the local dielectric landscape and the material average grain size, thus increasing the exciton binding energy and the spatially confinement of the excitons^24^, eventually leading to PLQY enhancement and to blue shift of the emission peak upon starch addition with respect to the reference. The presence of an insulating polymer, in addition, improves light extraction by reducing the refractive index or even enhancing the effective optical absorption by screening the charge at the polymer-perovskite interfaces. Finally, the better coverage of the substrate obtained with polymer improves the active interfaces leading to high current densities.

## Results

**Figure 1:**
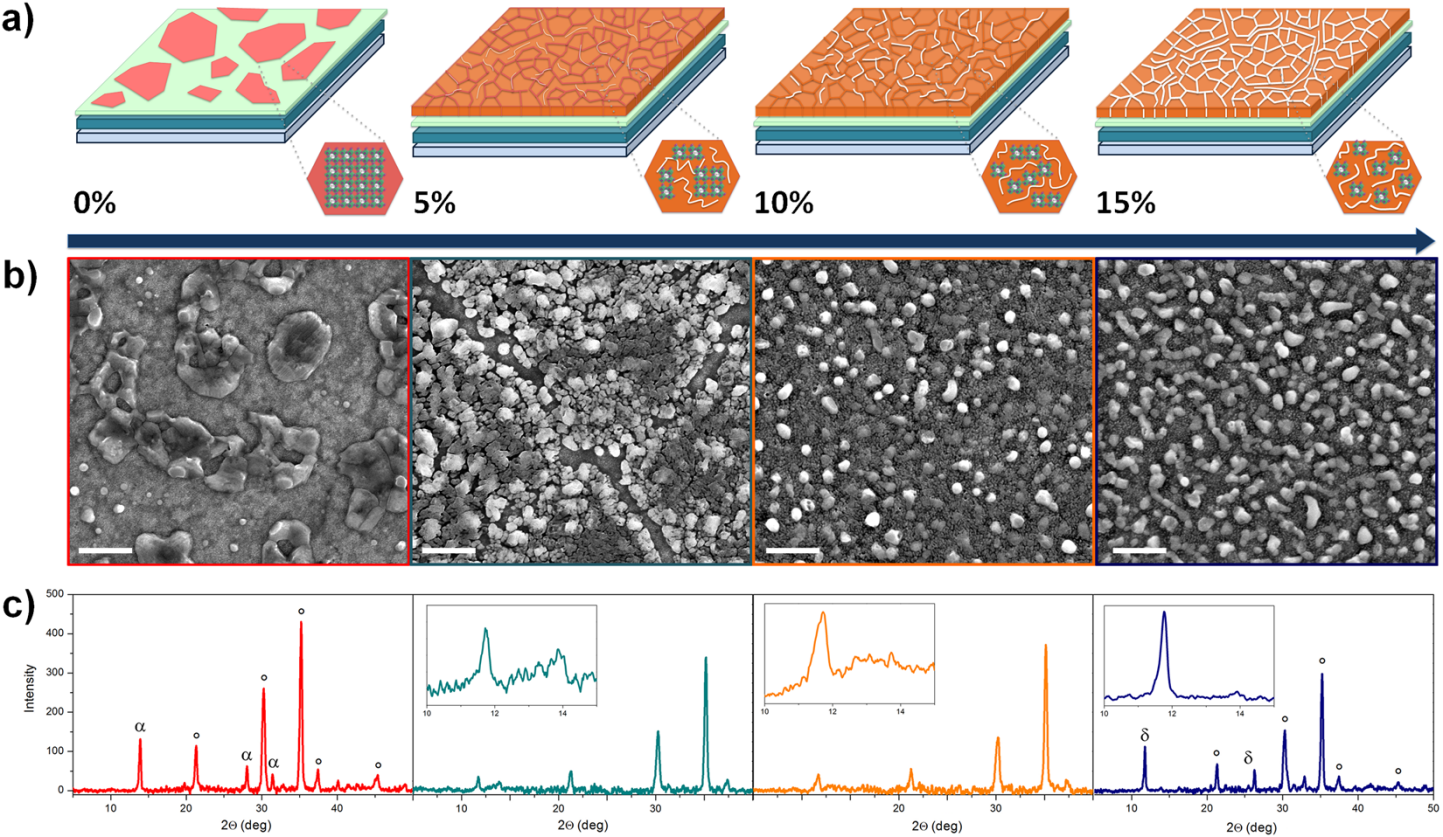
A cartoon representing the influence of the starch molecules on the perovskite film morphology and structure upon starch addition (**a**). SEM images of perovskite films fabricated by one step deposition of FAPbI_3_ precursors solutions mixed with different starch %wt. content (scale bar 1 μm) (**b**), XRD spectra of the respective films: α, δ and ° denote the identified diffraction peaks corresponding to the α and δ phases of FAPbI_3_ perovskite and ITO substrate, respectively (**c**).

Among the organic cations used as perovskite precursors, we have selected formamidinium (FAI) that forms a highly symmetrical perovskite structure with a band gap of 1.48 eV^25,26^. To promoted the formation of stabilized emissive α phase (from δ phase) at low annealing temperature, together with much enhanced NIR emission^24^ and resistance to humidity we introduce here an excess amount of FAI in the perovskite precursors (FAI: PbI_2_ = 2:1). FAPbI_3_ films were obtained by a single spin coating step of perovskite formulations with different starch biopolymer content onto polyethylenimine ethoxylated (PEIE) modified ZnO substrates. Film morphology was analyzed by scanning electron microscopy (SEM) and depicted in Figs 1(b) and S2. Partial and patchy film coverage, characterized by isolated and large grains (>1 µm) with irregular shapes were observed in the case of pristine FAPbI_3_ perovskite (Fig. 1(b)), resulting, as expected for this one-step unmodified process, in a large amount of the substrate remained uncovered. This inhomogeneous surface morphology and formation of pinholes in perovskite films would result in the formation of a bad interface with the transport layers and electrical shunt paths when integrated in the actual PeLEDs. Advantageously, by adding starch with increasing amount, perfect surface coverage was obtained and the perovskite morphology was enhanced reaching well-interconnected small grains with sizes down to 100 nm, as showed in Fig. 1(b). The improved surface coverage morphology is expected to enhance the charge injection and balance, reducing leakage currents in the resulting PeLEDs^27^.

Remarkably, the physical/chemical interaction between starch (Fig. S1) and perovskite precursors allowed the deposition of a smooth and homogeneous FAPbI_3_ film in a simple single step deposition^20^. The hydrogen chemical interaction with the –OH groups of the starch macromolecule^20^ can indeed interfere with the self-aggregation properties and the complex dynamic equilibria existing in solution between the perovskite precursors, profoundly affecting the morphology of the resulting polycrystalline film. Accordingly, we found that the size of the perovskite grains is strictly related to the concentration of starch, with the crystal size decreasing by increasing the starch content, as shown in Fig. 1(a).

The crystalline structure of pristine perovskite and with the addition of starch at different concentrations was examined by X-ray diffraction (XRD), as reported in Fig. 1(c). The XRD pattern of the pristine FAPbI_3_ film (red curve), showed a diffraction peak at 2θ = 13.9°(−110) and less intense diffraction peaks at 2θ = 28.1°(220) and 2θ = 31.5°(222), which corresponds to FAPbI_3_ α-phase^24,28^, confirming that the excess FAI contributed to the formation of α-FAPbI_3_ at mild annealing temperature (100 °C). The presence of 5% and 10% of starch into FAPbI_3_ leads to reduced intensity and broadening of the XRD peaks associated to the α-phase perovskite (inset Fig. 1(c) green and orange spectra), suggesting that the presence of biopolymer resulted in decreased crystal sizes^19^. In presence of starch, another peak appeared at 2θ = 11.8° (010), corresponding to δ-phase of FAPbI_3_^24,26^. In general, the variation of FAI precursors concentration affects the equilibrium between α and δ FAPbI_3_ phases, stabilizing one or the other, by acting on the colloid particle size and thus on the total free energy (including surface and bulk) of the two phases^24^. We found that the introduction of biopolymer led to the formation of a α-δ FAPbI_3_ mixed phase and the intensity of δ-phase peak was enhanced by increasing starch concentration, eventually resulting in well-defined δ-phase peaks at 2θ = 11.8° and 2θ = 26.1° and an almost total disappearance of the α-phase peak.

It is worth to underline that, because the thicknesses of the active perovskite films are very low, especially for the 5% and 10% being ~60 nm and ~80 nm thick respectively, the XRD signals are weak and the contribution of the substrate is remarkable. In Figure S3 we report the pristine starch as reference, showing no evidence of crystalline phases.

Thus, the inclusion of biopolymer led to a more complex crystalline final structure comprising mixed domains of α and δ FAPbI_3_ phases organized in grains with a reduced size homogeneously distributed on the substrate^20,21,29^. From FT-IR analysis (Fig. S4) of starch and perovskite/starch composites, we noticed a shift of the characteristic band of OH of starch that can be safely attributed to the hydrogen interaction between starch and perovskite precursors^20,30^. The starch in addition gelatinized in DMSO solvent creating a matrix, containing a large number of –OH groups, in which the perovskite grains are embedded during crystallization, a situation that affects the grain size.

Differential scanning calorimetry (DSC) measurements (Fig. S5) showed that starch modify the crystallization enthalpy of FAPbI_3_ perovskite, being 81.1 J/g for pristine perovskite and 58.9 J/g with the addition 5% of starch, likely due the establishment of hydrogen bonding between starch and FA (see FT-IR) already in the precursor solution^29^. This can promotes the formation of an increasing amount of δ-phase in FAPbI_3_, by incremental addition of starch.

The combined effect of the phase mixing, along with the physical confinement of such crystallite into a polymer matrix, had a dramatic effect on the performances of PeLEDs based on these composites.

**Figure 2:**
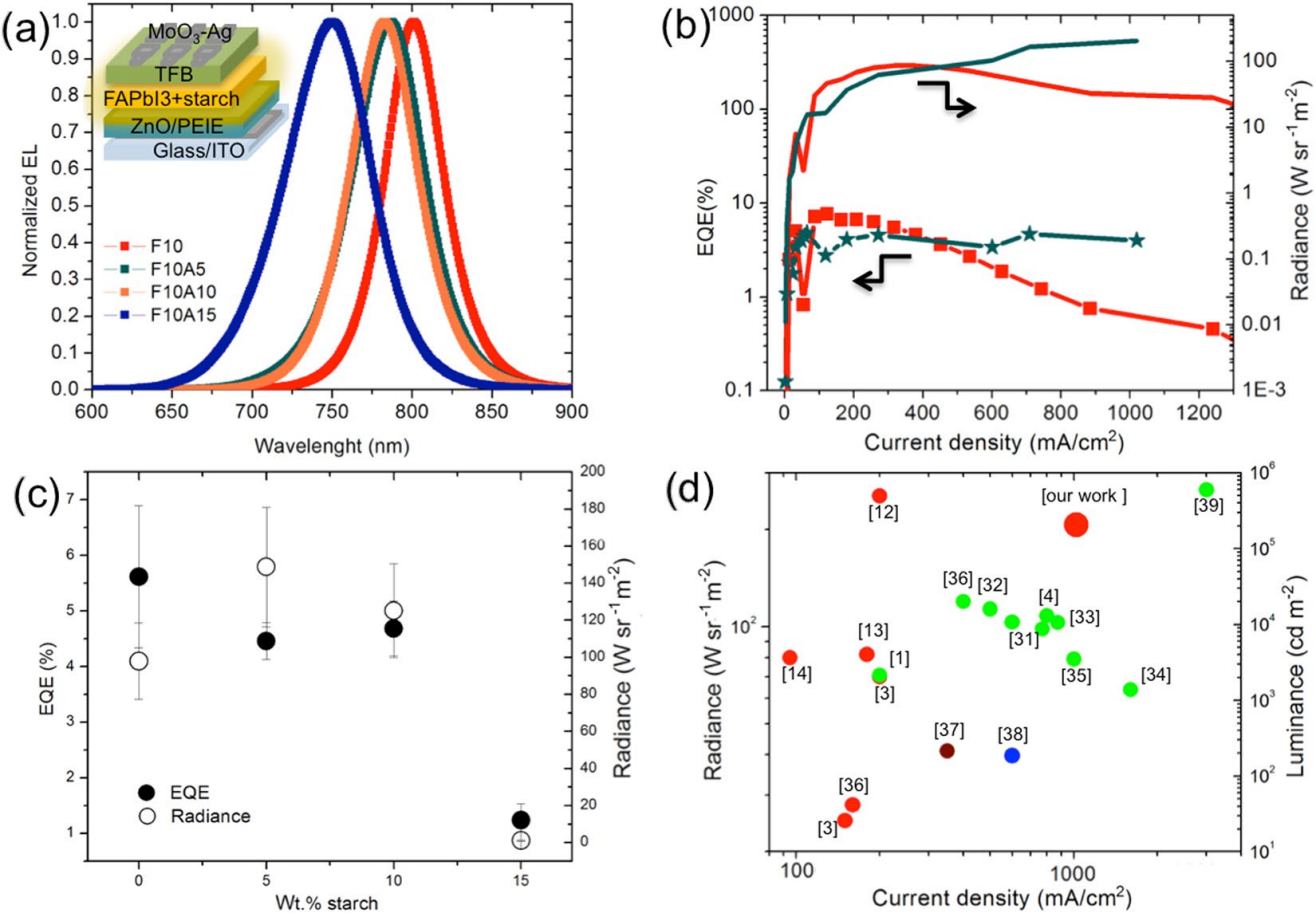
Normalized electroluminescence (EL) spectra of the devices with 0% (red), 5% (green), 10% (orange) and 15% (blue) of starch, recorded at maximum EQE voltages, the inset is the device architecture (**a**); Radiance (line) and EQE (line + simbols) vs Current density curves of the best device containing 0% and 5% of starch (**b**); Maximum EQE and radiance averaged on 16 LED devices as a function of starch concentrations (**c**); Radiance and Luminance versus current density of selected PeLEDs reported in literature (the numbers correspond to the references) (**d**)^1,3,4,12–14,31–39^.

The FAPbI_3_ films were integrated into devices consisting of multilayer stack, as schematically depicted in the inset of Fig. 2(a). The PEIE modified ZnO film was the electron transporting layer while the poly(9,9-dioctyl-fluorene-co-N-(4-butylphenyl)diphenylamine) (TFB) was used as hole transporting layer^13^. The normalized electroluminescence (EL) spectra of the devices (recorded at maximum EQE voltages) are reported in Fig. 2(a). The EL emission peak at the NIR, 801 nm, characteristic of 3D-FAPbI_3_ perovskites^13^, blue shifted by about 14 nm, 18 nm and 50 nm after adding 5%, 10% and 15% of starch respectively. This blueshift is a first clear indication of the perturbation caused by the starch mixture to the optical properties of the perovskite films. Such a blue shift can be correlated to the α/δ phase junction, causing a change in the dielectric constant of the media surrounding the emitting crystals. In our case, the biopolymer, thanks to its insulating nature, also contributes to dielectric landscape modification. The shape of EL spectra was not altered by the starch addition nor by different bias voltages applied up to reached max EQE. Only at high voltages the emission peak blueshifted by 6 nm, 5 nm and 4 nm in presence of 0%, 5% and 10% of starch respectively, while it stayed unaltered at the higher starch concentration (15%), as reported in Fig. S6. This observation suggests that increased starch concentration offers some protection to the perovskite active layer against detrimental effects related to high current densities.

The maximum EQEs and radiance of LED devices as a function of starch concentration, obtained by taking the maximum value for each LED devices and by calculating the standard deviation for each formulation, are reported in Fig. 2(c). The device without starch (named:F10) achieved a remarkable best EQE value of 7.7% at 2.4 V with a current density of 122.9 mA cm^−2^ and a radiance of 86 W m^−2^ sr^−1^. Noticeably the LED device containing 5% of starch reported in Fig. 2(b), showed a maximum EQE value of 4.8% at 2.8 V with a current density of 63.9 mA cm^−2^, but it reached an outstanding high radiance of around 206 W m^−2^ sr^−1^ at 4 V and at very high current density of 1020 mA cm^−2^. In panel b of Fig. 2, it is very clear the different behaviour between the referential F10 based device and the starch containing one. The reference gave overall better results in terms of EQE at low current regimes, while at higher currents a roll-off effect is evidently linked to a decrease of the performances (panel b), meanwhile the starch containing device showed an almost constant EQE (around 4.8%) up to 1000 mA cm^−2^, highlighting an excellent tolerance to high carrier densities regimes and a reduced efficiency roll off if compared to previous reports^12^. This is a very important result, as for most LEDs, the efficiencies diminished abruptly at high current densities, here, the presence of the starch allows keeping high-efficiency operation even at very high current levels. In addition, a very low turn-on voltage of 1.4 V was observed for the best device (starch 5%), even lower than the emission photon energy of 1.6 eV, suggesting thereby efficient and barrier-free charge injection into the perovskite emitter, as consequence of the improved interfaces. This, combined with the rapid increase of both current density and radiance, by orders of magnitude after turn-on, demonstrated a very low series resistance and efficient operations of the PeLED device^13^ as a clear evidence of the existence of a very good carrier percolation path through perovskite-interconnected domains among the insulating starch matrix and δ phase. The very high radiance would also depend from these considerations, as an easy injection and a smoother homogeneous film would allow reaching higher current withstanding no leakages nor shorts^6^. The absence of perturbation in the EL spectra (absence of blueshift in the sample containing 15% of starch), under incremental applied voltages, is attributed to an effective screening of the emitting crystals in relation to the electric field increase. Noticeably, the performances, in terms of radiances, currents and EQE, are among the highest reported in literature for PeLED device operating at near-infrared wavelengths, as showed in Figs 2(d) and S7^1,3,4,12–14,31–39^.

The effect of starch continued to raise perovskite performance up to a concentration of 10%, which achieved a high EQE of 5.5% at 2.8 V and a best radiance value of 170 W m^−2^ sr^−1^ at 4 V and 936 mA cm^−2^ of current density. However, the LED device containing 15% of starch showed a drastic reduction of performances down to 1.5% of EQE achieved at 2 V with a current density of 6.5 mA cm^−2^ and a radiance of 1.4 W m^−2^ sr^−1^ at 3.2 V and 33 mA cm^−2^. The highest starch concentration (15%) still brings the positive crystals size reduction and an improved coverage of the substrates, as seen in SEM analysis (Fig. 1(a)), however the insulating properties of the starch together with the formation of a higher concentration δ-phase, finally determined a high resistance to injected charges transport, as demonstrated by very low current density achieved if compared to the other devices.

Aiming at a further rationalize the starch influence on the material and device performances, we investigated the optical properties of the films. In perovskite materials, a range of different charge-carrier recombination mechanisms occur, which depend on the charge-carrier density (n) to a different degree. The rate of change in n can be expressed through the following equation (1):

1 $$\frac{{\rm{dn}}({\rm{t}})}{{\rm{dt}}}=-\,{{\rm{nk}}}_{{\rm{1}}}-{{\rm{n}}}^{{\rm{2}}}{{\rm{k}}}_{{\rm{2}}}-{{\rm{n}}}^{{\rm{3}}}{{\rm{k}}}_{{\rm{3}}}$$

where k_1_ is the monomolecular recombination rate constant (which is typically related to charge trapping), k_2_ is the bimolecular (electron–hole) recombination rate constant, and k_3_ is the third-order Auger rate constant. In the case of free carriers and a bulk perovskite, only the bimolecular recombination is associated to emission of a photon, therefore for light-emitting applications it is of paramount importance to reduce the impact of the trap mediated and of the Auger deactivation processes. Spatial confinement increases the local carrier density and therefore radiative recombination; in the extreme case of quantum confinement, when the dimensions of the perovskite grains decrease below the Bohr radius associated with electrons and holes, typically 10 nm, electrons and holes do not move independently anymore and the radiative recombination rate becomes monomolecular, typically improving emission efficiency at low carrier densities.

**Figure 3:**
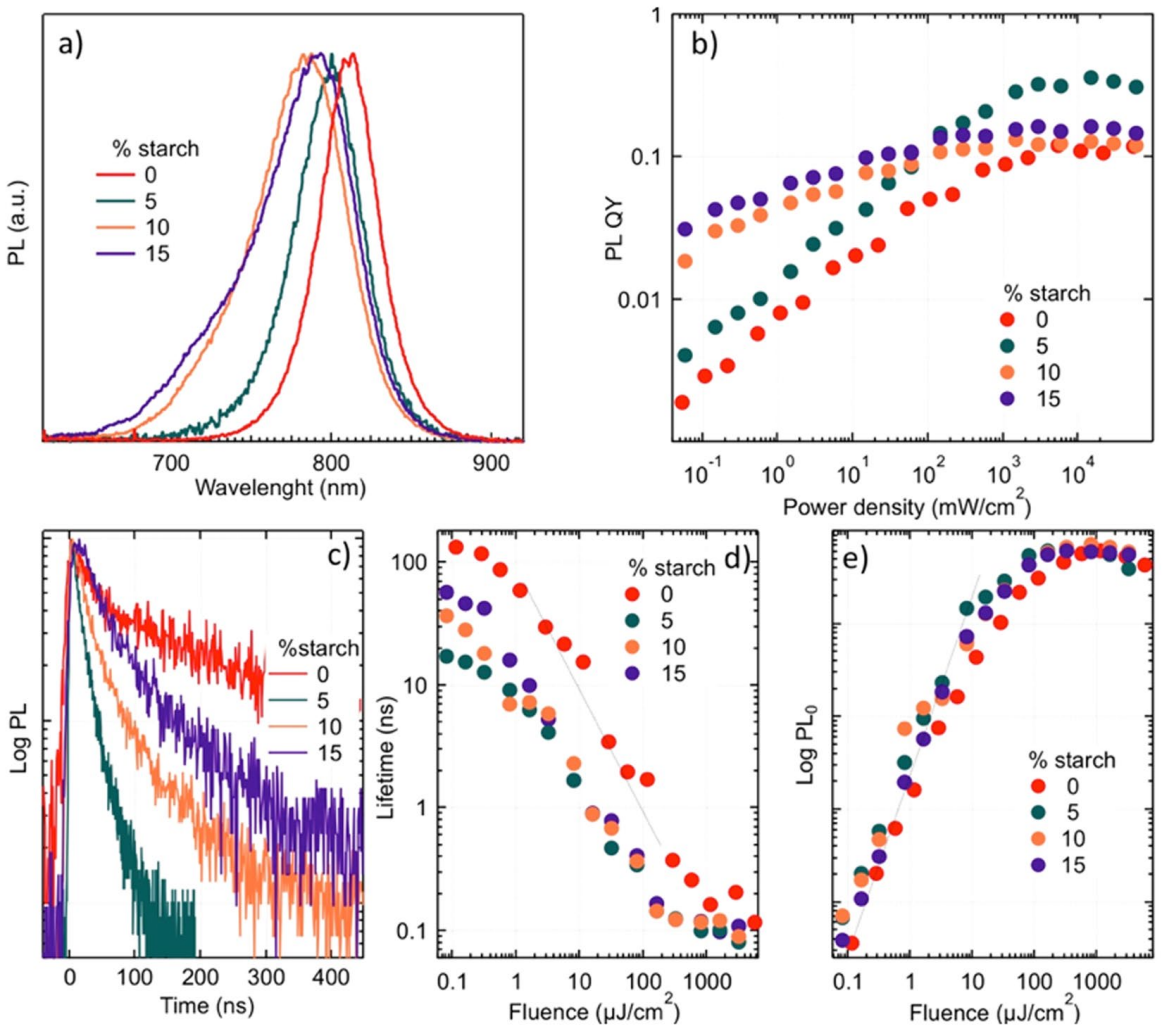
(**a**) PL spectra under low-intensity cw excitation (less than 1 mW/cm^2^). Inset: PLQY of the composites films. (**b**) Photoluminescence quantum yield (excitation wavelength 532 nm) as a function of the excitation density. (**c**) Temporal decays of PL measured at low excitation fluence. (**d**) PL lifetime (defined as 1/e decay time) extracted from photoluminescence decays as a function of the laser pulse fluence, present in log-log scale; the dotted line shows a linear dependence. (**e**) Instantaneous photoluminescence intensity *PL*_0_ measured as a function of laser pulses fluence and presented in log-log scale; the dotted line shows a quadratic dependence.

Photoluminescence (PL) measurements provided useful information to understand the effect of starch addition on radiative recombination processes. The steady-state PL measurements were carried out on FAPbI_3_ films, as a function of starch content; the spectra are showed in Fig. 3a. The PL emission peak of pristine FAPbI_3_ is located at 813 nm, in line with what reported for α-phase of this material^24^. The emission peak blue shifted by around 15 nm, 30 nm and 37 nm by adding 5%, 10% and 15% of starch, respectively. This observation confirmed the EL results and further underlined an effect related to the biopolymer interaction with perovskite formation. The emission quantum yield measures the efficiency of radiative recombination with respect to competing processes and, since in perovskite radiative recombination is bimolecular, its value tends to increase with carrier density. Figure 3b shows the PLQY of the perovskite films under continuous wavelength (cw) excitation with a green laser (532 nm in wavelength) of variable intensity. The addition of starch improved the emission efficiency with respect to the reference, pure perovskite sample, both at low excitation density, where the largest starch concentrations led to better performance, and at high excitation, where 5% starch concentration produced the best quantum yield. The temporal decay of photoluminescence under femtosecond pulsed excitation was measured with a streak camera in all samples, as shown in Fig. 3c. The addition of starch reduced the photoluminescence lifetime at low excitation densities, meaning that it enhanced trap-assisted recombination and its corresponding k1 coefficient, which should have reduced, not enhanced, the emitted PL intensity. The analysis of the PL lifetimes (Fig. 3d) showed that decay times decreased linearly with the pulse fluence (dotted line), as predicted for bimolecular recombination; at the highest fluences decay times were very close to the temporal resolution of the streak camera apparatus.

To rationalize how, in the starch-perovskite composites, the improvement in cw PLQY coexisted with the increase in non-radiative recombination rate, we measured the instantaneous PL intensity, PL_0_, emitted in coincidence with the excitation laser pulse (Fig. 3e). Such quantity, in the backscattering geometry, can be written as ${{\rm{PL}}}_{{\rm{0}}}=\frac{{{\rm{\Omega }}}_{{\rm{ext}}}}{{{\rm{n}}}_{{\rm{ref}}}^{{\rm{2}}}}\frac{{{\rm{k}}}_{{\rm{2}}}{{\rm{n}}}_{{\rm{0}}}^{{\rm{2}}}}{2{\rm{\alpha }}({{\rm{h}}{\rm{\nu }}}_{{\rm{exc}}})+{\rm{\alpha }}({{\rm{h}}{\rm{\nu }}}_{{\rm{em}}})}(1-{\rm{r}}),$ where ${{\rm{\Omega }}}_{{\rm{ext}}}$ is the solid angle of photoluminescence collection, ${{\rm{n}}}_{{\rm{ref}}}$ the hybrid perovskite refractive index, ${\rm{\alpha }}$ the absorption coefficient, ${{\rm{\nu }}}_{{\rm{em}},{\rm{exc}}}\,\,$the optical emission and excitation frequencies, ${\rm{r}}$ the sample reflectivity and ${{\rm{n}}}_{0}={\rm{\alpha }}({{\rm{h}}{\rm{\nu }}}_{{\rm{exc}}})\frac{{\rm{\Phi }}}{{{\rm{h}}{\rm{\nu }}}_{{\rm{exc}}}}$ the photocarrier concentration injected by each laser pulse of fluence ${\rm{\Phi }}$ ^40,41^. The main take-away from Fig. 3e is that PL_0_ was higher in samples with starch than without, meaning that the biopolymer either enhanced the radiative recombination coefficient k_2_ or improved light extraction by reducing the refractive index, or even enhanced the effective optical absorption because of dielectric scattering at the polymer-perovskite interfaces. In addition, the α/δ phase junction would also contribute to these phenomena. Importantly, the presence of starch did not alter the order of recombination dynamics, since for all samples the intensity of PL_0_ grew as the square of the pulse fluence (dotted line in Fig. 3e), which is the signature of emission by bimolecular recombination. At the highest available fluences, Auger recombination caused a saturation of PL_0_ at comparable values for all samples, an indication that the Auger coefficient k_3_ was not significantly affected by the presence of the biopolymer.

Combining morphological, structural and photophysical characterization, the overall picture that emerged is that the efficiency roll off is reduced and radiance is enhanced with starch thanks to a combiation of factors: *i)* the formation of α/δ mixed phase, assisted by starch, which could improve the carrier recombination in the perovskite layer avoiding losses at the interface with the hole/electron transporting layers^42^; *i)* improved substrate coverage of the perovskite film, which hinders current leakage; *iii)* modification of dielectric landscape at the starch-perovskite and at the α-δ interfaces, eventually enhancing the electron-hole radiative recombination or light out diffusion.

## Conclusion

In summary, by growing FAPbI_3_ perovskite crystals into a biopolymeric matrix we achieved control on the grain sizes and on the peculiar crystalline composition. A mixed α/δ phase junction, characterized by enhanced NIR emission, reduced grains size and α phase stabilization (from δ phase) at low annealing temperature, was in fact formed into the starch network. The use of starch controls the perovskite grain size, their electronic screening at high-carrier densities as well as the substrate coverage, ensuring high performing PeLEDs. The composites embedded in LED lead to maximum radiances above 200 Wm^−2^ sr^−1^ and very high efficiencies ~5% EQE obtained at high current densitiy regimes for a minimized efficiency roll-off effect.

## Methods

### Materials

Lead (II) iodide PbI_2_ ultradry 99.999% (metals basis) was purchased from Alfa Aesar, Formamidinium iodide HC(NH_2_)_2_I (FAI) from Xi’an Polymer Light Technology Corp. Corn starch (Maizena) was purchased from Unilever, DMSO was purchased from Fisher BioReagents. 2-isopropanol (HPLC, 99.9%), ethanol (anhydrous, 99.8%), Polyethylenimine (PEIE) 80% ethoxylated solution, Zinc acetate dihydrate (99.999%), were all purchased from Sigma Aldrich.

Poly[(9,9-dioctylfluorenyl-2,7-diyl)-co-(4,4′-(N-(4-sec-butylphenyl)diphenylamine)] (TFB) was supplied from American Dye source Inc. M-xylene 99% extra pure was purchased from Acros Organics. All the materials were used as received without any further purification. ZnO nanocrystals was prepared as previous reported^33^. ITO substrates were purchased from Shenzhen Huayu Union Technology Co. Ltd.

### Starch-perovskite precursors solution preparation

The perovskite precursors solution with a modified precursors stoichiometry, i.e. FAI: PbI_2_ = 2:1, were prepared by mixing FAI with PbI_2_ in DMSO with a concentration of 10 wt% respect to the solvent. The solution was stirred at 80 °C for 30 min. After precursors solubilisation, different starch/precursors ratios were added to each solution, i.e. 0 wt%, 5 wt% 10 wt% and 15 wt%, that were stirred at 80 °C for 5 h in order to obtain a clear solution after starch solubilisation.

### Morphological Characterization

Scanning electron microscope images of the starch-perovskite composite samples, deposited on ITO/ZnO-PEIE modified substrate at 7000 rpm for 100 s and annealed at 100 °C for 10 min, were collected by using Carl Zeiss Auriga40 Crossbeam instrument, in high vacuum and high-resolution acquisition mode, equipped with Gemini column and an integrated high efficiency in-lens detector. The applied acceleration voltage was 5 kV.

### X-ray diffraction (XRD) analysis

Wide-Angle X-ray diffraction spectra of FAPbI_3_ film containing different starch concentrations, deposited on ITO/ZnO-PEIE modified substrate at 7000 rpm for 100 s and annealed at 100 °C for 10 min, were collected on a PW 1729 Philips, using Cu Kα radiation in reflection mode (λ = 0.154 nm). All the samples were step-scanned at room temperature from 2θ values of 5°− 40°.

### DSC Analysis

Dynamic DSC scans were performed starch-perovskite precursor solutions, without and with 5 wt% of starch/precursors ratio, by a differential scanning calorimeter (DSC Mettler Toledo 622). About 10 μL of liquid samples was put into opened aluminium flat disks and heated from 25 up to 200 °C at 10 °C min^−1^ scan rate under nitrogen atmosphere flow at 60 mL min^−1^. The endotherm related to solvent evaporation has been subtracted in order to isolate the perovskite crystallization exotherm.

### FT-IR analyses

Infrared spectra were recorded on starch film and FAPbI3 film with and without starch, deposited on silicon substrate at 7000 rpm for 100 s and annealed at 100 °C for 10 s. The analyses were conducted in the wavelength range between 4,000 and 700 cm^2^, using a Fourier Transform Infrared spectrometer (FTIR) Jasco 6300. Each measurement was obtained with 128 scans and 4 cm^2^ of resolution.

### Device Fabrication and Testing

ITO coated glass substrates were treated with detergent overnight and then by TL1 (NH_3_: H_2_O_2_: H_2_O = 1:1:5) procedure for 20 min. ZnO nanoparticles were deposited in ambient condition by spin coating at 4000 rpm for 40 s and annealed at 150 °C for 30 mins. PEIE dispersed in IPA solution with a concentration of 0.05 wt% was spin coated on the top of ZnO at 5000 rpm for 40 s. Perovskite based solutions, with and without starch, were spin-coated on ITO/ZnO/PEIE in glovebox at 7000 rpm for 100 s and then the films were annealed at 100 °C for 10 min. TFB solution (8 mg/ml in m-xylene) was spin-coated onto the perovskite film. Finally, 10 nm of MoO_3_ film and 100 nm of Ag film were deposited as electrode inside of the thermal evaporator.

The performances of all the Perovskite LED devices were measured in a nitrogen-filled glovebox at room temperature. A Keithley 2400 was used to collect current density and driving voltage data and an integrating sphere together with the Go pro spectrometer (Ocean Optics) were used to collect emission data. The device working area is 0.0725 cm^2^.

### Photoluminescence and quantum yield

In order to measure continuous-wave photoluminescence spectra, samples were excited with a diode-pumped Nd:YVO_4_ CW laser (Spectra Physics Millennia V) at 532 nm. The photoluminescence was dispersed with a grating spectrometer (Princeton Instruments Acton SpectraPro 2500i equipped with a 150 gr/mm, 600 nm blaze grating) and detected by a LN-cooled CCD camera (Princeton Instruments). The absolute photoluminescence quantum yield was calibrated using an Edinburgh FLS920 spectrometer equipped with a Peltier-cooled Hamamatsu R928 photomultiplier tube (185–850 nm). An Edinburgh Xe900 450 W xenon arc lamp was used as exciting light source. Corrected spectra were obtained via a calibration curve supplied with the instrument. Lamp power in the QY experiments about 0.6 mWcm^−2^, spot area 0.5 cm^2^.

QY have been determined by using a barium sulphate coated integrating sphere (4 or 6 inches), following the procedure described by de Mello *et al*.^43^.

In time-resolved photoluminescence experiments, samples were excited with a regenerative amplified laser (Coherent Libra) delivering 130-fs-long pulses at a repetition rate of 1 KHz. Photoluminescence was dispersed with a grating spectrometer (Princeton Instruments Acton SpectraPro 2300i equipped with a 50 gr/mm grating blazed at 600 nm), dispersed and detected by a streak camera (Hamamatsu).

## Electronic supplementary material

Supplementary Information
