# Supplementary material for: Ultra-Bright Near-Infrared Perovskite Light-Emitting Diodes with Reduced Efficiency Roll-off
Source: Sci Rep. 2018 Oct 19;8:15496. doi: 10.1038/s41598-018-33729-9 (PMC6195535; doi:10.1038/s41598-018-33729-9)

Supporting Information

Ultra-Bright Near-Infrared Perovskite Light-Emitting Diodes with Reduced Efficiency Roll-off

Antonella Giuri[1,2], Zhongcheng Yuan[3], Yanfeng Miao[4], Jianpu Wang[4], Feng Gao[3], Nicola Sestu[5], Michele Saba[5], Giovanni Bongiovanni[5], Silvia Colella[1,6], Carola Esposito Corcione[2], Giuseppe Gigli[1], Andrea Listorti[1,6], and Aurora Rizzo[1]**

1. Istituto di Nanotecnologia CNR-Nanotec, Distretto Tecnologico via Arnesano 16, 73100 Lecce, Italy

2. Dipartimento di Ingegneria dell'Innovazione, Università del Salento, Via per Arnesano, 73100 Lecce, Italy

3. Biomolecular and Organic Electronics, IFM, Linköping University, 58183, Linköping, Sweden

4. Key Laboratory of Flexible Electronics (KLOFE) & Institute of Advanced Materials (IAM), Jiangsu National Synergetic Innovation Center for Advanced Materials (SICAM), Nanjing Tech University (Nanjing Tech), 30 South Puzhu Road, Nanjing 211816, China.

5. Dipartimento di Fisica, Università degli Studi di Cagliari, I-09042 Monserrato, Italy

6. Dipartimento di Matematica e Fisica "E. De Giorgi", Università del Salento, Via per Arnesano, 73100 Lecce, Italy

Figure S1 Corn Starch molecular structure, based on repeating α-D-glucopyranosyl units organized into linear amylose (about 27%) and branched amylopectin (about 73%)

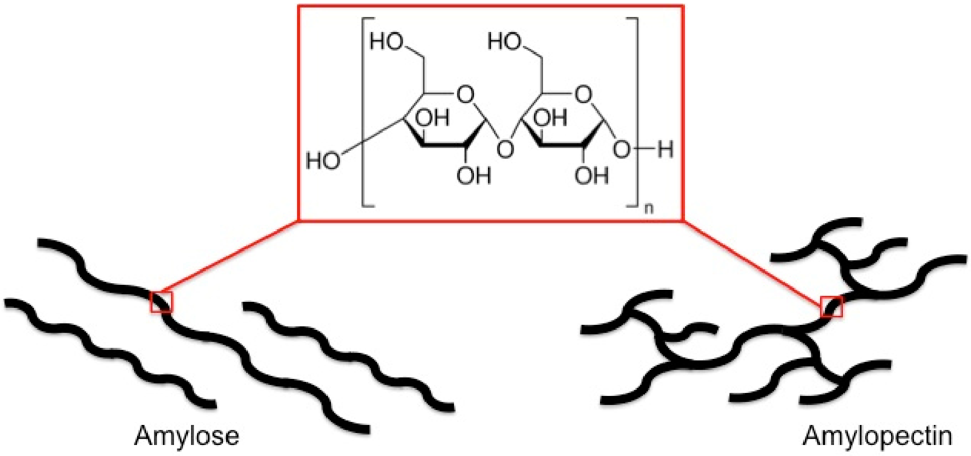

Figure S2. SEM images of perovskite films fabricated by one-step deposition of $FAPbI_3$ precursor solutions mixed with different starch %wt. content: (a) 0%, (b) 5%, (c) 10%, (d) 15%. Scale bars are 1 μm for left column and 200 nm for right column images.

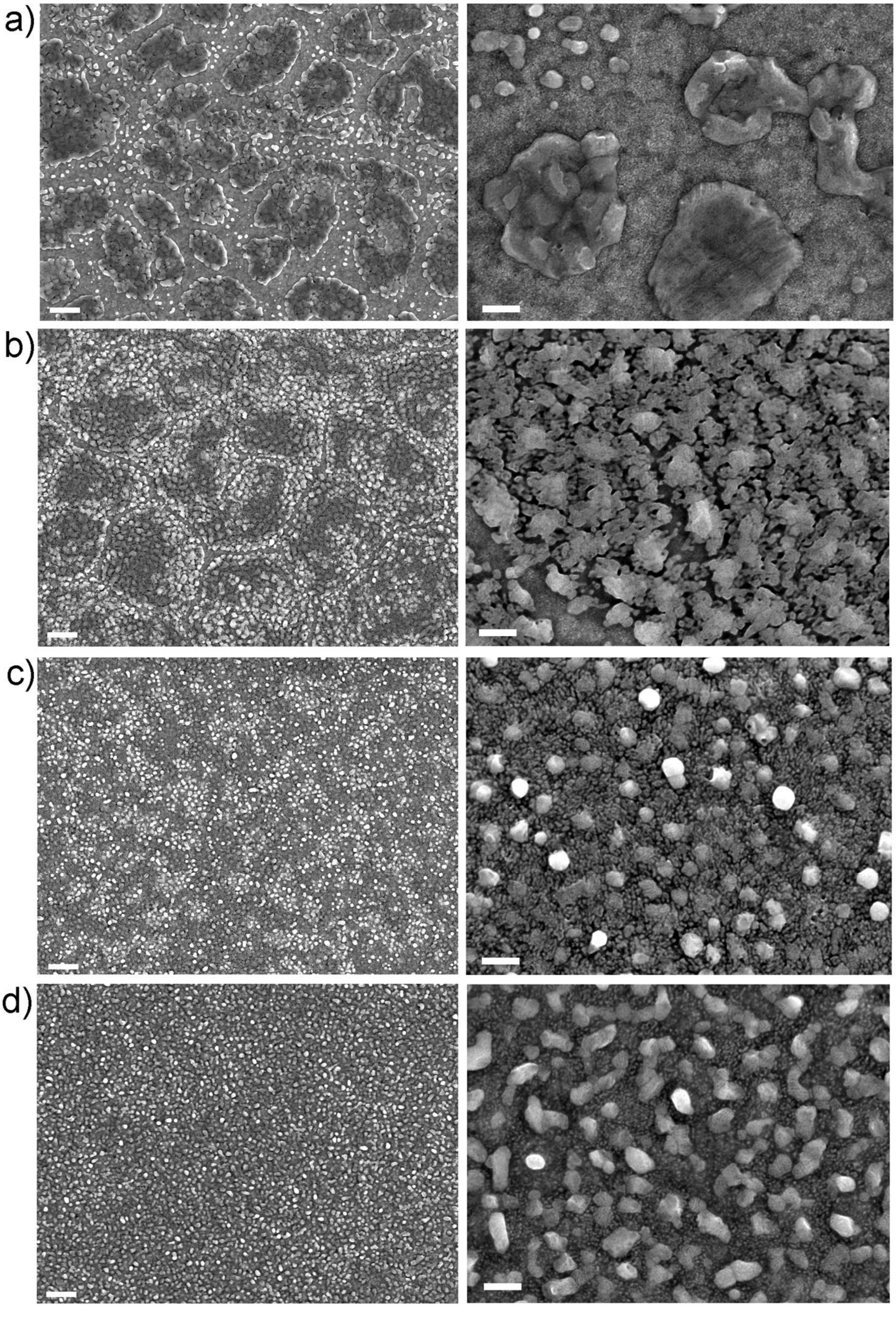

Figure S3. XRD spectra of a starch biopolymer film without perovskite, deposited on glass substrates from DMSO solvent. In the inset a zoom in the region of interest.

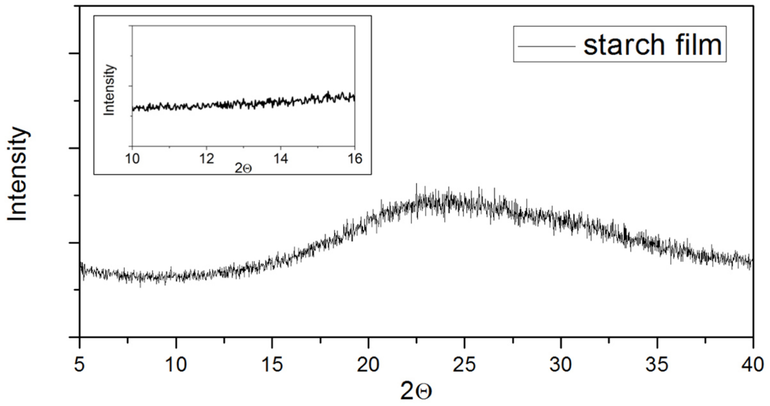

Figure S4. Fourier Transformed Infrared Spectra of F10 reference perovskite film (red) of the starch (green) and of a blend of perovskite and 5% starch (black).

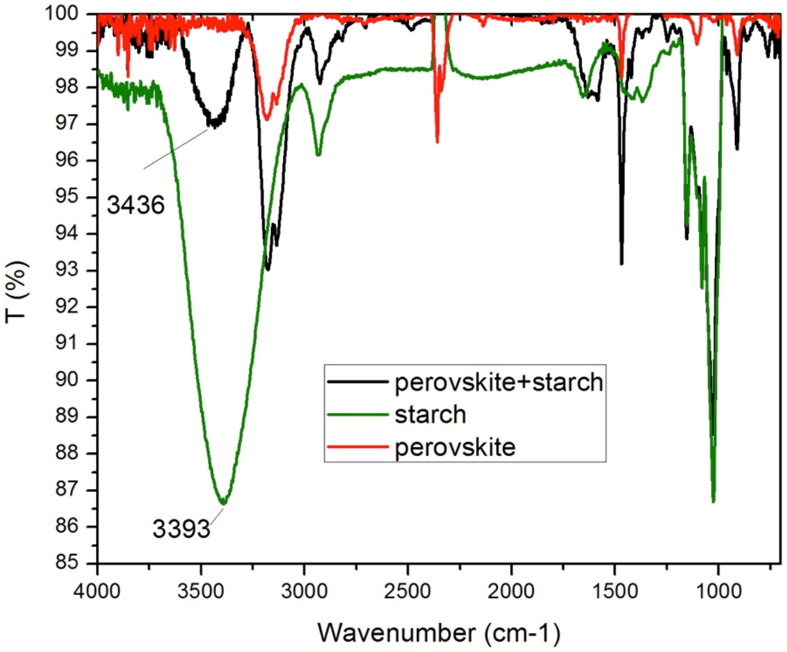

Figure S5. Differential scanning calorimetry (DSC) measurements of reference perovskite (red) and perovskite with the addition of 5% of starch (green) formulations.

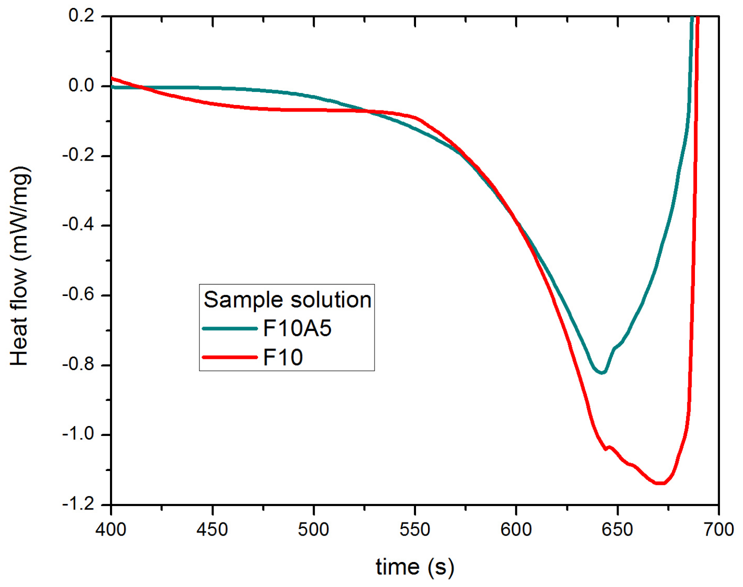

Figure S6 and TableS1. Normalized electroluminescence (EL) spectra and relative values (TableS1) of the devices with 0%(panel a), 5%(panel b), 10%(panel c) and 15% (panel d) of starch, recorded at different increasing voltages, in the inset of Figure 1a is reported the device architecture(a).

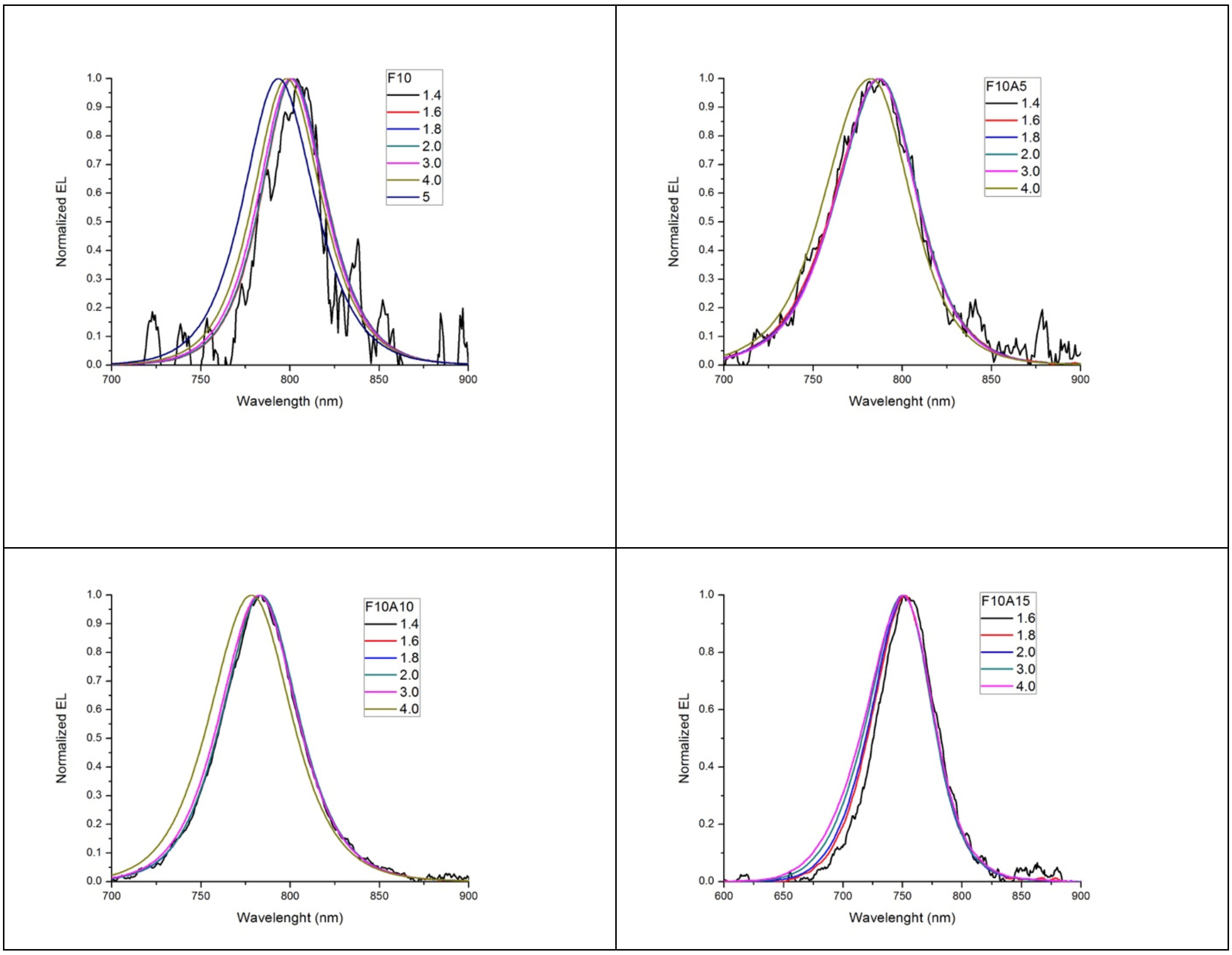

	F10		F10A5		F10A10		F10A15	
Applied V (V)	Peak (nm)	EL Intensity	Peak (nm)	EL Intensity	Peak (nm)	EL Intensity	Peak (nm)	EL Intensity
1.4	804	0.0018	788	0.00437	783	0.018		
1.6	802	0.7667	787	0.1345	783	0.5394	751	0.01174
1.8	801	3.5807	788	0.6069	783	1.1331	751	0.06675
2	801	1.002	787	0.7685	783	5.6043	751	0.1781
3	800	35.9907	787	6.1606	783	36.1072	751	0.4266
4	798	25.2962	783	73.2774	779	63.4082	751	0.2579

Figure S7. EQE versus current density of selected PeLED reported in literature (the numbers correspond to the references in the main text of the manuscript)

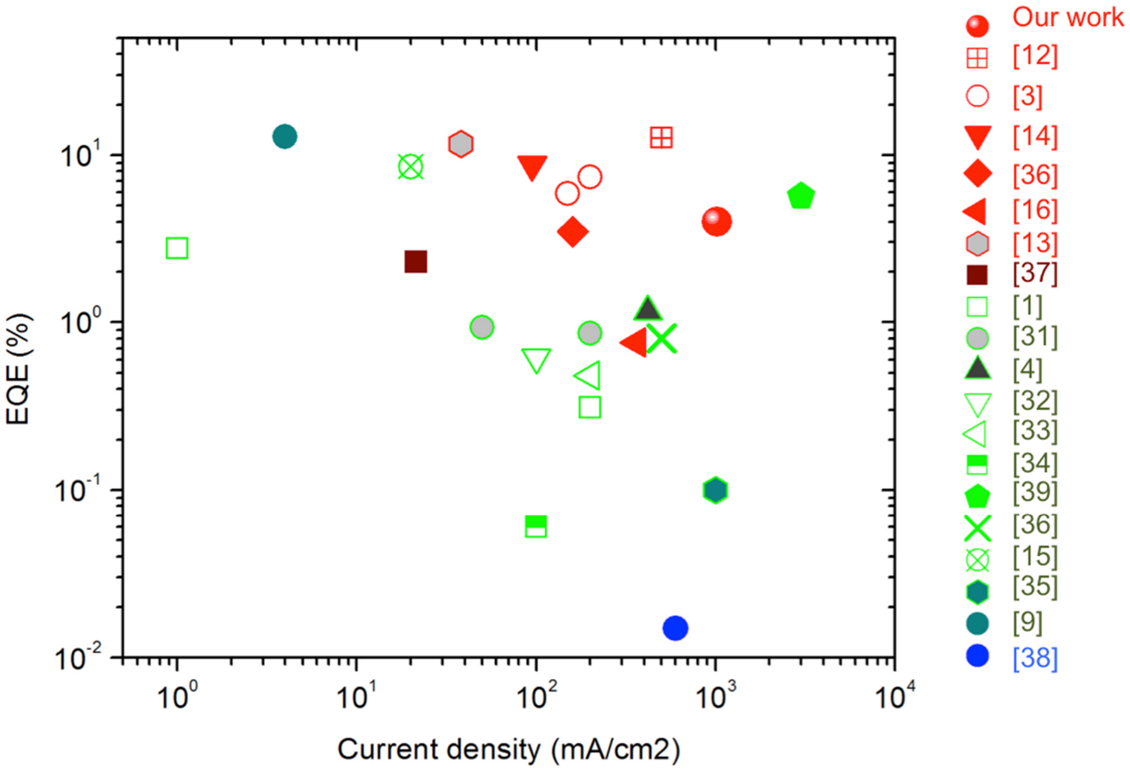

Supplement: Supplementary file 1 — Supplementary Information [file 41598_2018_33729_MOESM1_ESM.pdf]
